# Plastic Surgery

**Published:** 1917-05-05

**Authors:** 


### PLASTIC SURGERY.

Plastic surgery is divisible into grafting or the repair of soft parts—as in the case of hare-lip or the removal of unsightly scars—and prosthesis or the restoration of mutilated parts. Both branches have been in use for long periods of time; prosthesis, perhaps, is the more ancient as it was employed amongst nations where mutilation was a recognised punishment. It has come down to us as the Indian operation, and its first great modification was made in 1597 by Tagliacozzi, the Italian surgeon, immortalised in "Hudibras" as:—

> So learned Taliacotius from,
> The brawny part of porter's bum
> Cut supplemental noses which
> Would last as long as parent breech.

There was, however, but little change until Reverdin in 1869 and Thiersch, a few years later, introduced an extended system of skin grafting. Modern improvements in technique, combined with more cleanly methods in surgery, showed that organs could be transplanted successfully and enabled marvellous advances to be made in connection with the surgery of the blood-vessels. Prosthesis underwent no similar change, and it is only within the last few months that it has begun to come into its own heritage. The advance has been made in consequence of the disfiguring wounds of the war, and in this country the work of Captain H. D. Gillies, Mr. Percival Cole, and Captain L. A. B. King have revolutionised the methods and have established principles which must prove of the utmost value for a still further advance. In the case of severe deformities of the face it has been found advantageous for the surgeon to work with the dentist, as most of the more serious injuries involve the bones as well as the soft parts. There is thus developing a form of dental surgery which needs not only special manipulative skill, but also a very consummate knowledge of bone surgery. It is clear that rest and repair of the bone must be secured before any attempt is made to improve the appearance of the patient, whilst in every case the restoration of function is of more real importance than the cosmetic effect. Infinite patience, minute attention to detail, and the reduction of septic inflammation to a minimum are the key-notes to successful treatment, and it is clear that the more the same surgeon and dentist work together, the better are the results.

Many injuries can be treated, but there are still some which are beyond the reach of surgery. For these Lieutenant Derwent Wood manufactures cunningly modelled shields which are tinted to represent the natural colours of the skin. The shields are worn in such a manner that they effectually conceal the deformity, and are yet easily removable for cleaning purposes. By their use a man with a greatly disfigured face will not only pass unnoticed in a crowd but will bear comparatively close inspection.

The results obtained from plastic surgery are most encouraging, but it is evident that with greater experience much greater advances will be made. This, however, can only be done by entrusting the special hospitals devoted to the reception of face and jaw injuries to the care of those who have shown themselves specially fitted for the work. These men should be relieved of all administrative work and should be given rank in recognition of the pioneer work which they are doing. They are at present captains and lieutenants, they should be at least majors.

